# Almond Consumption and Inflammatory Biomarkers: A Need for Further Investigation

**DOI:** 10.1016/j.advnut.2023.03.013

**Published:** 2023-04-03

**Authors:** Pavlo Petakh, Anatolii Lohoida, Aleksandr Kamyshnyi

**Affiliations:** 1 Department of Microbiology, Virology, and Immunology, I. Horbachevsky Ternopil National Medical University, Ternopil, Ukraine; 2 Department of Biochemistry and Pharmacology, Uzhhorod National University, Uzhhorod, Ukraine

Dear Editor

We are writing in response to the recent article published in your journal, titled “The Effects of Almond Consumption on Inflammatory Biomarkers in Adults: A Systematic Review and Meta-Analysis of Randomized Clinical Trials” [1]. The study aimed to summarize the current literature on whether almonds can affect inflammatory markers, and the authors concluded that almond consumption has a beneficial effect on CRP and IL-6 concentrations in adults but has no notable effects on TNF-α, ICAM-1, or vascular cell adhesion molecule-1 (VCAM-1).

We find this study to be of great interest because inflammation is a key driver of many chronic diseases, including CVD and type 2 diabetes [2]. Almonds are a rich source of nutrients, including fiber, protein, and healthy fats, and have been previously shown to have beneficial effects on cardiovascular health [3]. Therefore, understanding the effects of almond consumption on inflammation is important in developing dietary recommendations for disease prevention [4].

However, we would like to raise some concerns regarding the methodology of the study. Firstly, the authors included studies with a wide range of almond doses, ranging from 10 to 113 g/d. The beneficial effect of almond intake on inflammatory biomarkers only occurred at doses <60 g/d, which suggests a dose–response relationship. Therefore, the heterogeneity in almond doses across studies may have contributed to the mixed results on inflammatory biomarkers. Secondly, the subgroup analyses showed that the effects of almond consumption on CRP and IL-6 were nonsignificant in unhealthy participants or in those with obesity. This suggests that the effects of almonds on inflammatory biomarkers may be dependent on baseline health status or body weight, and therefore, further studies are needed to explore these relationships.

In conclusion, the study provides valuable insights into the effects of almond consumption on inflammatory biomarkers in adults. However, further studies are needed to explore the dose–response relationship and the effects of almonds on inflammation in different population groups.

## Conflict of Interest

The authors declared no potential conflicts of interest concerning the research, authorship, and/or publication of this article.
